# A carbohydrate-active enzyme (CAZy) profile links successful metabolic specialization of *Prevotella* to its abundance in gut microbiota

**DOI:** 10.1038/s41598-020-69241-2

**Published:** 2020-07-24

**Authors:** Juhani Aakko, Sami Pietilä, Raine Toivonen, Anne Rokka, Kati Mokkala, Kirsi Laitinen, Laura Elo, Arno Hänninen

**Affiliations:** 1 Turku Bioscience Centre, University of Turku and Åbo Akademy University, Turku, Finland; 2 Institute of Biomedicine, University of Turku, Medisiina D 7022, Kiinamyllynkatu 10, 20520 Turku, Finland; 3 Department of Clinical Microbiology and Immunology, Turku University Hospital, Turku, Finland

**Keywords:** Classification and taxonomy, Databases, Proteome informatics, Computational biology and bioinformatics, Microbial communities, Metabolism

## Abstract

Gut microbiota participates in diverse metabolic and homeostatic functions related to health and well-being. Its composition varies between individuals, and depends on factors related to host and microbial communities, which need to adapt to utilize various nutrients present in gut environment. We profiled fecal microbiota in 63 healthy adult individuals using metaproteomics, and focused on microbial CAZy (carbohydrate-active) enzymes involved in glycan foraging. We identified two distinct CAZy profiles, one with many *Bacteroides-*derived CAZy in more than one-third of subjects (n = 25), and it associated with high abundance of *Bacteroides* in most subjects. In a smaller subset of donors (n = 8) with dietary parameters similar to others, microbiota showed intense expression of *Prevotella*-derived CAZy including exo-beta-(1,4)-xylanase, xylan-1,4-beta-xylosidase, alpha-l-arabinofuranosidase and several other CAZy belonging to glycosyl hydrolase families involved in digestion of complex plant-derived polysaccharides. This associated invariably with high abundance of *Prevotella* in gut microbiota, while in subjects with lower abundance of *Prevotella*, microbiota showed no *Prevotella-*derived CAZy. Identification of *Bacteroides-* and *Prevotella-*derived CAZy in microbiota proteome and their association with differences in microbiota composition are in evidence of individual variation in metabolic specialization of gut microbes affecting their colonizing competence.

## Introduction

Human gut microbiota has evolved to live in commensalism and in beneficial mutualism with the host. It consists of hundreds of microbial species, which collectively function to provide the host protection against pathogens, augment digestion of nutrients, synthesize vitamins and stimulate immune system^1^. Besides many aspects of health and diseases, variations in its composition associate with differences in dietary preferences and body mass index^2,3^. A low *Bacteroidetes*-to-*Firmicutes* ratio and high prevalence of *Faecalibacterium* associate with high dietary energy intake and overweight, whereas high representation of *Bacteroides*-species in microbiota associates with diets high in fat and animal-derived protein content; and although not consistently, according to some studies it associates with a higher risk to develop metabolic disease^4^. Diets poised towards plant-derived foods often favor *Prevotella* abundance in microbiota. Much of this variation in microbiota depends on differences in metabolic competence of individual microbes adapted to forage different nutrients available in the gut milieu, namely complex polysaccharides of plant hemicelluloses and pectin, animal-derived glycans and host mucus^5^.

PUL (polysaccharide utilization loci) are gene clusters organized around *susC* and *susD* (starch utilization system) genes in bacteria^6^. PUL encode multiprotein complexes responsible for binding structurally complex polysaccharides to outer membrane of bacteria and for initiation of their depolymerisation^7,8^. A single PUL may contain various numbers of enzymes (carbohydrate active enzymes, CAZy) required for initial degradation of plant-derived complex polysaccharides or animal glycans (plant fiber and animal glycan degraders), or degradation of the arising less complex glycans (secondary degraders). Degradation of complex polysaccharides requires many steps and a large number of CAZy, which are often organized to several PUL, as recently characterized for rhamnogalactonuronan II degradation in *Bacteroides thetaiotamicron*^9,10^. Species of the genus *Prevotella* have also been identified to encode similar PULs with glycosyl hydrolases (GH) and other CAZy. Profiling of CAZy genes present in *Prevotella*-isolates representing 50 different species originally isolated from gut, oral cavity or rumen revealed robust differences in their abilities to degrade animal- and/or plant-derived glycans^11^. Thus, *Prevotella* have adapted to degrade glycans from different sources at least on species-level, and apparently depending on their preferred habitat in humans and cattle.

In metagenomic profiling of carbohydrate-active enzymes (CAZy) in gut microbiotas, *Prevotella*-annotated CAZy genes differed between individuals, and this associated with microbiota response to diet modification^12^. Although several important aspects of fecal microbiota and gut homeostasis have been addressed by proteome analyses^13–17^, CAZy in fecal microbiota have so far been analyzed only on a few occasions on genomic^12^ and transcriptomic^18^ level. In this study, we studied CAZy in fecal microbiota on whole proteome level by newly designed analysis tools^19,20^, and related their CAZy expression to their microbiota composition. We found individual differences between donors, as the expression of various CAZy clustered into several groups of CAZy co-occurring more likely together in same samples. We also identified two distinct profiles among members of gut microbiota in association with high or low abundance of *Bacteroides* in microbiota. Enhanced expression of CAZy participating in metabolism of plant cell-wall –derived glycans in a few donors and its association with high abundance of *Prevotella* in their microbiota suggests exceptional metabolic competence of *Prevotella* present in some donors to utilize particular carbohydrates including complex plant-derived glycans.

## Results

### Gut microbiota proteomes identify proteins derived from various commensals of human gut microbiota

**Figure 1:**
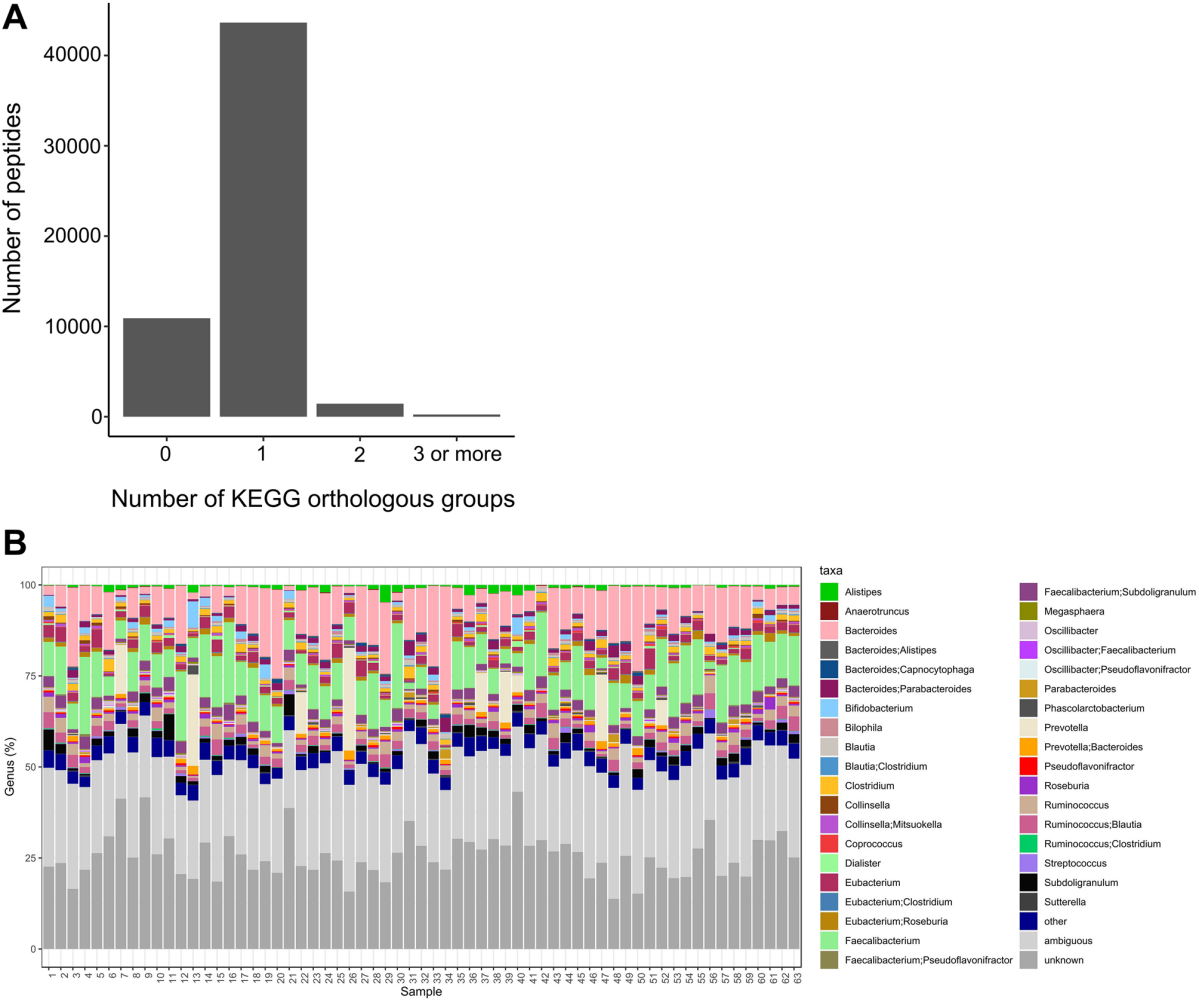
Numbers of functional (**A**) and taxonomical (**B**) annotation of the identified peptides. (**A**) Most of the identified peptides belong to a single orthologous group. (**B**) At genus level, appr. 20–25% of peptides were unanimously annotated to one bacterial genus, while appr. 40–60% of peptides was shared by two or more genera. Peptides remaining without taxonomic annotation constituted 13.8–43.2% of peptides. (For a particular genus, see color codes).

Metaproteomes of gut microbiota extracted from fecal samples were analyzed by mass spectrometry and searched against protein databases. Samples were from a cohort of overweight and obese female donors (n = 63) derived from a dietary intervention study ^21^ and taken at entry to the study (see Suppl. Table 1 for donor characteristics). A single KEGG orthologous group annotation was assigned to 43,636 peptides (Fig. 1), which represented 1,446 different KEGG orthologous groups. Furthermore, 1,989 peptides annotated to two or more orthologous groups, while 10,901 remained unannotated. The total number of peptides identified ranged from 5,415 to 17,904 peptides per sample (median 11,868 peptides/sample).

Genus level taxonomical annotations were assigned to the peptides by utilizing taxonomic annotations of the integrated reference catalog of the human microbiota^16^. Depending on individual microbiota, one particular genus was assigned to 14.6–27.5% of the peptides. A further 38.5–59.0% of peptides were listed ambiguous between two taxa, reflecting their origin from either of two defined bacterial genera (Fig. 1), while 13.8–43.2% of peptides remained without a taxonomic annotation.

Depending on the donor, three different genera dominated the gut metaproteome. In the majority of donors, most of the genus-specific peptides were annotated either as *Bacteroides* or *Faecalibacterium* –specific peptides. A few donors were distinguishable by a high proportion of *Prevotella*-specific peptides in their microbiota. Genus-specific annotations included also many other genera including *Bifidobacterium, Ruminococcus, Alistipes, Clostridium, Collinsella* and *Parabacteroides* (Fig. 1).

### Glycoside hydrolases and other bacterial CAZy co-occur in groups according to their bacterial annotations

**Figure 2:**
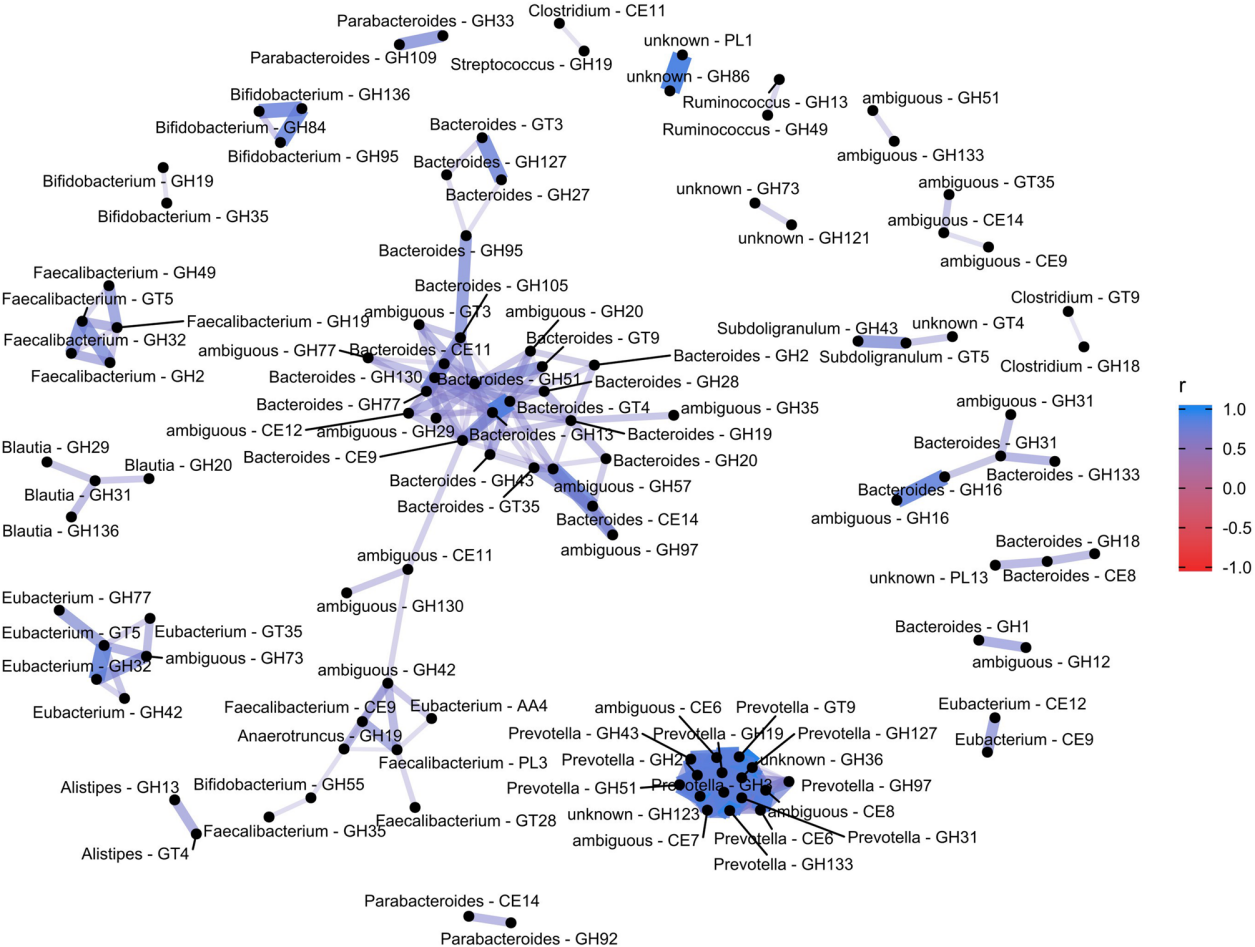
Co-occurrence network of CAZy families of particular bacterial genera in the samples. *Bacteroides*- and *Prevotella*-derived glycosyl hydrolases (GH) cluster together in distinct sets of samples. Clusters containing fewer GH enzymes can be identified for *Faecalibacterium*, *Eubacterium* and *Bifidobacterium*- derived enzymes. Each node represents a genus–CAZy family pair, and the edges represent a significant co-occurrence relationship (see text for details). The top 500 correlating pairs were included in the graph.

CAZy enzymes are organized in families of glycoside hydrolases (GH), polysaccharide lyases (PL), carbohydrate esterases (CE) and glycosyl transferases (GT) according to sequence homology with one or more biochemically characterized enzymes. Glycosyl hydrolases are the most diverse group of enzymes, currently divided to 162 families. Correlation analysis of CAZy enzymes distinguished several clusters of glycosyl hydrolases that co-occurred in the samples. Most notable were a cluster formed by *Bacteroides*-annotated glycosyl hydrolases, and another cluster formed by *Prevotella*-annotated glycosyl hydrolases (Fig. 2). In addition, a few smaller clusters of glycosyl hydrolases with genus-level annotations were identified, including those annotated to *Faecalibacterium*, *Bifidobacterium* and *Eubacterium*.

### Abundance of *Bacteroides* in microbiota associates with expression of *Bacteroides*-derived CAZy enzymes

**Figure 3:**
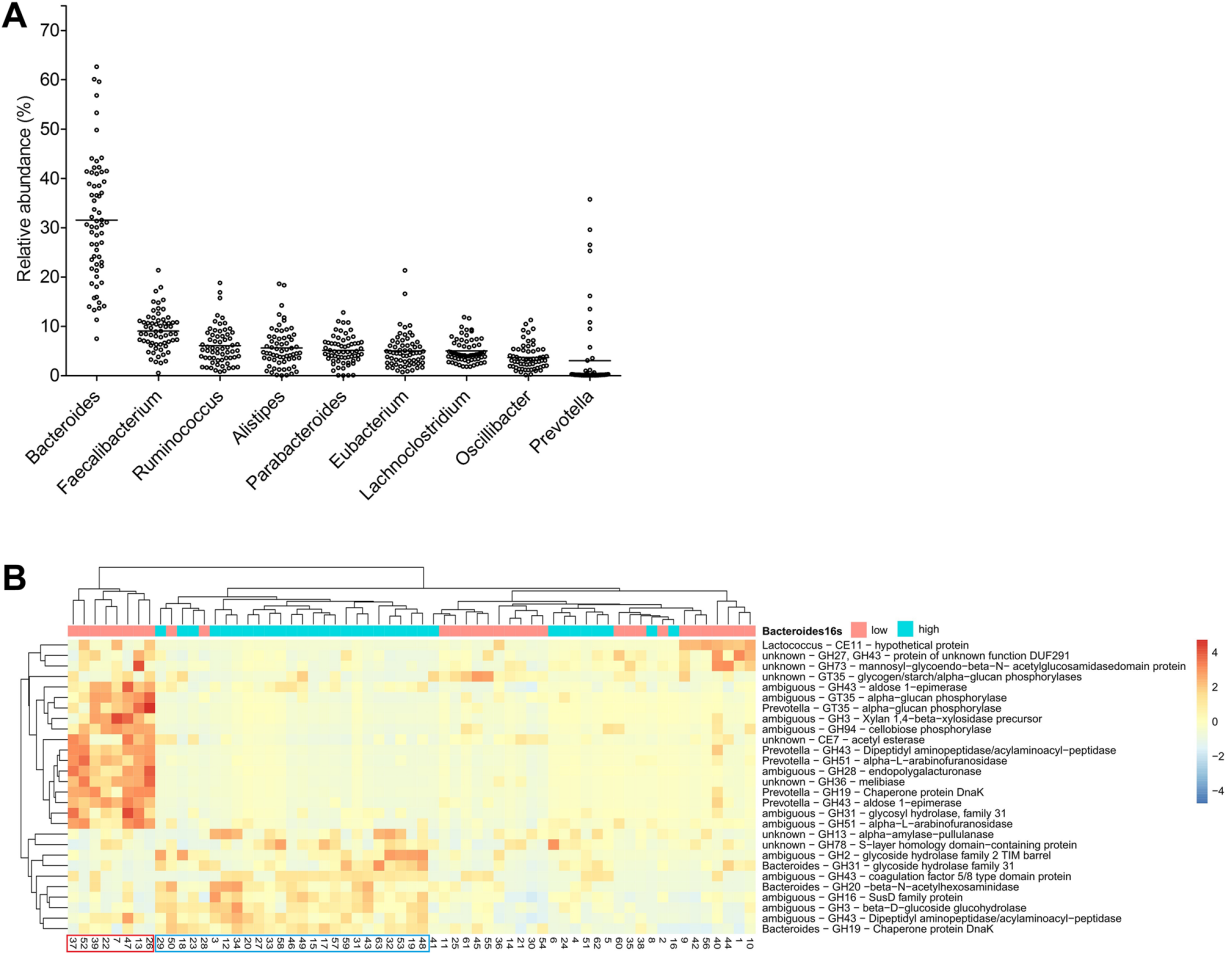
Donors with high abundancy of *Bacteroides* and donors with high abundance of *Prevotella* differ in their expression of CAZy. (**A**) Relative abundance of the most prominent bacterial groups on genus-level according to 16S-RNA parallel sequencing. (**B**) A heatmap of CAZy enzymes identifies two distinct profiles of CAZy enzyme expression among donors (in vertical columns). CAZy enzymes (horizontal lines) annotated as *Bacteroides*- or *Prevotella*-derived (or ambiguous) are expressed at a higher level in two distinct groups of donors (consisting of 25 and 8 donors, demarcated with blue or red frames, respectively). In (**A**), each dot represents the value of abundance for one donor. Enzymes with a FDR < 0.01 are included in the heatmap**.**

Typical to microbiota in developed countries, *Bacteroides* was the most abundant genus among our donors. According to 16S-RNA gene sequencing results, *Bacteroides*-genus constituted between 7 and 62% (median 35%) of the whole microbiota (Fig. 3A). To identify individual CAZy annotations and their potential association with *Bacteroides* abundance in microbiota, we compared CAZy expression between donors with high abundance (above median) of *Bacteroides* to donors, in which *Bacteroides* represented a less prominent proportion of whole microbiota. This comparison identified 25 donors, whose microbiota proteome expressed a number of *Bacteroides*-derived CAZy enzymes (Fig. 3B and Suppl. Figure 1). This proteome pattern associated with high abundance of *Bacteroides* in most donors, although not all donors showing high abundance (above median) of *Bacteroides* in microbiota showed this CAZy pattern either at a false discovery rate (FDR) < 0.01 or < 0.05 (Fig. 3B and Suppl. Figure 1).

According to 16S rRNA gene sequencing results, the abundance of *Bacteroides* in microbiota correlated negatively with several other genera, most strongly with *Ruminococcus, Faecalibacterium,* and *Prevotella,* but also to *Alistipes* and *Oscillibacter* (not shown). Similar to samples with high *Bacteroides* abundance, we profiled samples with high abundance of *Faecalibacterium* as the second most abundant genus in our donors (see Fig. 3A) against all other samples. Although well over one hundred CAZy were differentially expressed between individual samples, donors with high abundance of *Faecalibacterium* in their microbiota did not present any CAZy profile distinct from other donors (not shown).

**Figure 4:**
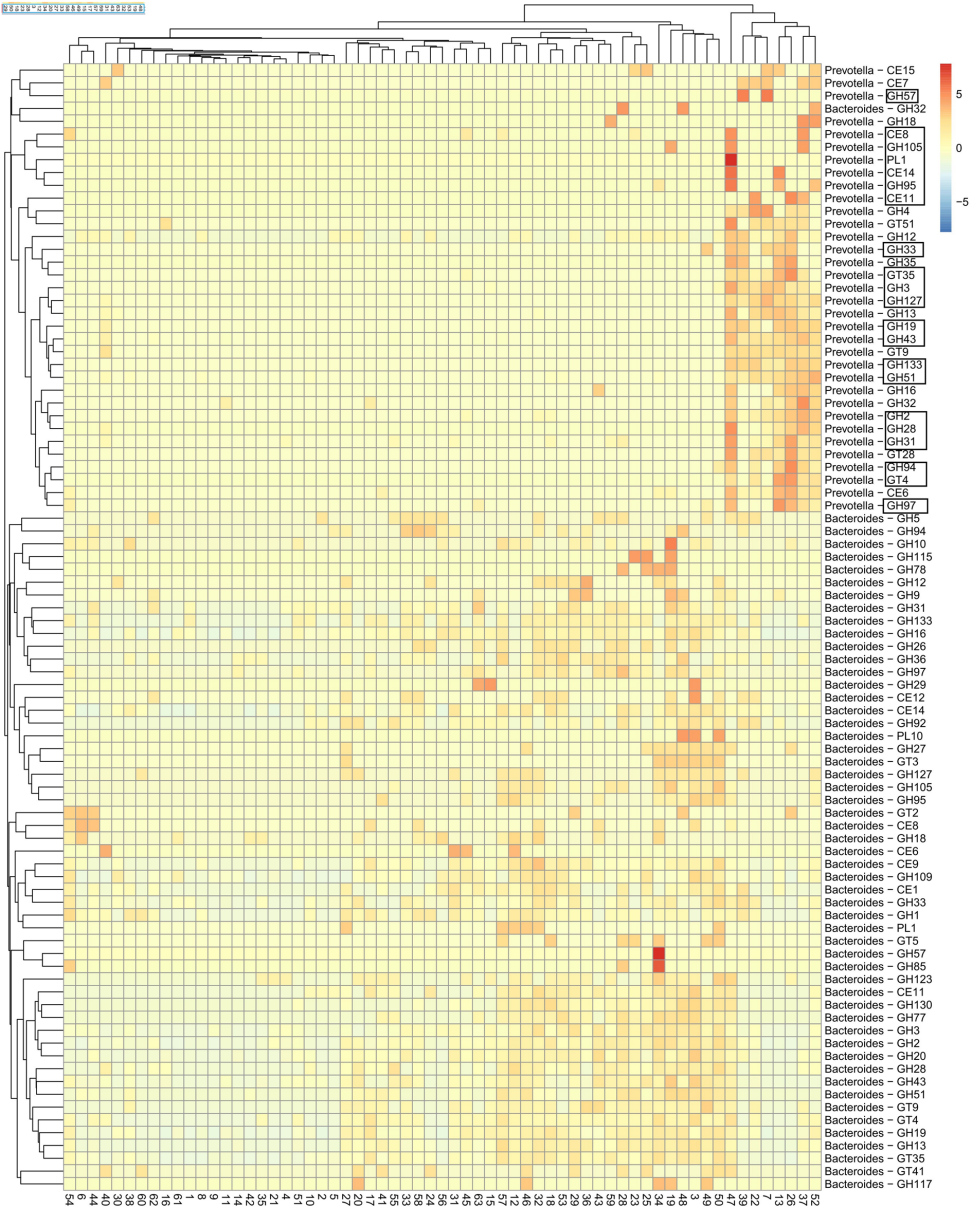
(**A**) A list of glycosyl hydrolase (GH) and other CAZy families with unambiguous *Prevotella* or *Bacteroides*-annotation in the form of a heatmap. Of the 88 families found in this heatmap (horizontal lines), 21 are shared (framed with black for their *Prevotella-*annotated identifications) and appear in both clusters and groups of donors. Inclusion of CAZy families is based purely on peptide annotations without any profiling of donors.

**Figure 5:**
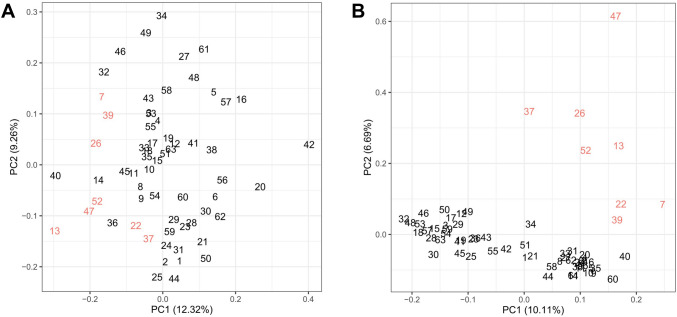
*Prevotella* representation in microbiota of study subjects and its relationship to *Prevotella*-CAZy proteome. (**A**) Principal component analysis (PCA) of microbiota composition based on 16S-RNA sequencing at genus level and (**B**) based on intensity-weighed CAZy enzyme expression. In both PCA-plots, donors with high abundance of *Prevotella* (red) differ significantly from all others (black) according to permutational multivariate analysis of variance (PERMANOVA) (p < 0.001 and p < 0.01, for (**A**) and (**B**), respectively). Numbers refer to the number of each donor in heatmaps (Figs. 3B, 4).

Profiling of donors with *Bacteroides*-dominated microbiota against other donors made also another CAZy profile apparent. A cluster of CAZy enzymes, most of which were annotated as *Prevotella*-derived enzymes, was expressed in 8 donors (13% of all donors) more intensely compared to other donors (Fig. 3). We next profiled expression of all CAZy families annotated as *Prevotella* and *Bacteroides* to compare CAZy expression between *Bacteroides* and *Prevotella*. Without setting any prior selection criteria, we retrieved 88 CAZy families, of which 54 were *Bacteroides-* and 34 *Prevotella* -annotated, organizing donors in *Bacteroides-* and *Prevotella* clusters as before (Fig. 4). Of these CAZy families, *Bacteroides* and *Prevotella* shared only 21 CAZy families, suggesting that *Prevotella* and *Bacteroides* express partially distinct enzymes. We then profiled *Bacteroides* and *Prevotella*-annotated CAZy on the level of enzyme name annotations, and identified 13 enzymes belonging to these shared 21 CAZy families. Of these 13 enzymes in shared CAZy families, only 3 were the same enzymes, while 10 were different (Suppl. Figure 2). Thus, *Prevotella* and *Bacteroides*-CAZy were partially distinct in our donors, suggesting metabolic specialization of *Prevotella* in individuals with high abundance of *Prevotella* in their microbiota. Donors with high abundance of *Prevotella* differed in their microbiota composition (based on 16S rRNA gene parallel sequencing) as well as CAZy enzyme composition compared to other donors (PERMANOVA, p < 0.001 and p < 0.01, respectively) Thus, a unique microbiota composition in these donors associated with a unique CAZy profile (Fig. 5).

### *Bacteroides* and *Prevotella* CAZy-profiles suggest adaptation to metabolism of different substrates

Enzymes identified in *Bacteroides* and *Prevotella* CAZy profiles suggest differences in their substrate specificity. *Prevotella* CAZy profile contained several enzymes with predicted activities in metabolism of xylan and other complex polysaccharides derived from plants, such as GH51 alpha-l-arabinofuranosidase, GH28-family endopolygalacturonase, GH43 Beta-xylosidase and GH3 Xylan-1,4-beta-xylosidase (Suppl. Figure 1 and Suppl. Figure 2). This profile also included a CAZy belonging to GH13 family involved in digestion of resistant starch and glycogen. Alpha-amylase, alpha-glucosidase and beta-fructofuranosidase identified in this CAZy profile also suggest activities involved in hydrolysis of disaccharides and starch.

Compared with *Prevotella* CAZy profile, enzymes identified within *Bacteroides* CAZy profile suggested a different spectrum of specificities. Annotations in the latter included enzymes belonging to glycosyl hydrolase families GH18, GH20 and GH92, which contain enzymes active against animal glycans^22^, and a SusD family protein belonging to GH16, which contains CAZymes of various substrate specificities (Suppl. Figure 1 and Suppl. Figure 2). Enzymes such as, GH3 beta-d-glucosidase, GH2 beta-galactosidase and aldose-1-epimerase are involved in breakdown of oligo- and disaccharides ().

After identifying a number of CAZy highly expressed in *Prevotella*-rich microbiotas we performed a pathway analysis for enzymes identified in this enzyme cluster using their KEGG-ortholog codes^23–25^ and GOMixer pathway analysis (https://www.raeslab.org/omixer/visualisation/map). Accordingly, a number of enzymes in this CAZy cluster are involved in degradation of complex plant-derived polysaccharides and several of them in pectin degradation pathways, including 5-dehydro-4-deoxy-d-glucuronase and endopolygalacturonase (Table 1) and many in xylan degradation pathways. Other degradation pathways in which enzymes of this *Prevotella* CAZy cluster participate include starch, lactose, galactose and melibiose degradation pathways.

**Table 1:** Degradation pathways related to enzymes in the *Prevotella* CAZy-cluster. KEGG ortholog codes^23–25^ were analyzed with GOMixer pathway analyzer. See text for details.

Module ID	Module name	Orthologs
MF0064	Pectin degradation	K01051
		K01184
		K01213
MF0065	Pectin degradation-5-dehydro-4-deoxy-d-glucuronate degradation	K01728
		K01730
		K01815
		K00874
		K01625
MF0091	Beta-d-glucuronide and d-glucuronate degradation	K00040
		K00874
		K01195
		K01686
		K01812
MF0071	d-Galacturonate degradation	K01812
		K00041
		K01685
		K00874
MF0062	Starch degradation	K01176
		K01200
		K01187
MF0047	Lactose and galactose degradation (PTS)	K02786 + K02787 + K02788
		K01220
		K01819
		K00917
		K01635
		K08302
MF0048	Lactose degradation	K01190
MF0066	Glycogen metabolism	K00975
		K00703
		K00700
		K00688
		K01187
		K00705
MF0056	Galactose degradation (Leloir pathway)	K01785
		K00849
		K00965
		K01784
		K01838
MF0050	Melibiose degradation	K07407
MF0003	Acetylglucosamine degradation	K00884
		K01443
		K02564
MF0093	Homoacetogenesis (acetate production)	K05299 + K15022
		K01938
		K01491
		K00297
		K15023
		K00191 K00192 + K14138 K00196 + K14138 K00198 + K14138 K03518 + K03519 + K03520 + K14138
MF002	Ethanol production (CO_2_ pathway)	K01568
		K00001 K00121 K04072 K11440 K13953 K13954

### High abundance of *Prevotella* does not correlate with increased intake of fibers or other dietary factors

High abundance of *Prevotella* associates typically with diets rich in plant-derived glycans and dietary fibers. We therefore compared dietary parameters calculated from recorded food diaries. The overall dietary quality^26^ did not differ between donors displaying *Prevotella-*CAZy profile and donors not displaying this CAZy profile, yet having intermediate levels of *Prevotella* in microbiota (P = 0.397). However, when inspecting the individual IDQ (index of diet quality)^26^ food components reflecting the intake of fiber, the frequency of whole grain intake was in fact lower in individuals with *Prevotella-*CAZy profile, while there were no differences in frequency of vegetable or fruit consumption between the two groups (Table 2). We also compared energy intake and intake of various dietary components but found no differences in the diet to explain high abundancy of *Prevotella* in the group with *Prevotella*-CAZy profile (Suppl. Table 2).

**Table 2:** Consumption of whole grains, vegetables, fruits and berries among donors with exceptionally high abundance of *Prevotella* (and *Prevotella-*associated CAZy profile, left) and among donors with intermediate levels (and no CAZy profile, right) of *Prevotella* in their microbiota (median ± interquartile range, IQR). ^a^Index of diet quality (see text); ^b^the number of days consumed in a week.

	*Prevotella* with CAZy n = 8		*Prevotella* without CAZy n = 7		P-value
	Median	IQR	Median	IQR	P-value/Mann–Whitney *U* test
IDQ^a^	9.3	6.3–11.8	10	9.0–13.0	0.397
Whole grains^b^	5	4.0–6.8	7	7.0–7.0	0.040
Vegetables^b^	6.5	6.0–7.0	7	7.0–7.0	0.232
Fruits and berries^b^	5.5	5.0–7.0	7	3.0–7.0	0.779

## Discussion

The knowledge on CAZymes and their role in metabolic activities of gut microbiota is rapidly increasing based on genomic^11,12,18^, structural^6^ and biochemical studies^10^. The CAZy database (https://www.cazy.org) dedicated to compilation of this knowledge makes an important contribution to advance the understanding of the biological significance of carbohydrate-degradation potential in microbiota^27,28^. To extend the information provided by genomic analyses, these enzymes in microbiota need to be studied also on the level of transcriptome^29^, proteome and metabolome^30^, and using biochemical assays^31^. In this study, we utilized mass spectrometry coupled with a customized set of data analysis tools to characterize the CAZy expression of gut microbiota at proteome level from stool samples from a cohort of overweight (average BMI 30.2) but otherwise healthy females at their early pregnancy^21,32^.

In CAZy database (https://www.CAZy.org), enzymes participating in degradation of carbohydrates are organized into families of glycosyl hydrolases (GH), polysaccharide lyases (PL) and carbohydrate esterases (CE), in which individual enzymes are defined by sequences that cluster around one or a few biochemically characterized members. Although enzymes within these families vary in their substrate specificities^31^, the known functions of enzymes within many GH families are often restricted to one or a few types of carbohydrates, thus allowing the prediction of substrate specificity on a general level^22,33^. The spectral libraries generated from donor microbiotas allowed us to characterize the gut metaproteome to a level sufficient for identification of a number of CAZy and to link many of them to a particular bacterial genus. We identified CAZy across phyla and many bacterial genera. Although the methodological approach of purifying bacteria before protein isolation favors relative yields of cytoplasmic over cell-surface associated proteins^34^, we chose this approach on the basis of better overall yields of bacteria-associated proteins^34^. In addition to cytoplasmic CAZy, we identified among *Prevotella*-annotated CAZy some cell-surface associated proteins including a SusD family CAZy. Many of the CAZy were unanimously identified only on GH family level, but a number of CAZy in *Bacteroides* and *Prevotella* CAZy profiles were identified even by their actual enzyme names. According to these annotations, enzymes in both the *Bacteroides* and *Prevotella* CAZy profiles were predicted to have activities against complex polysaccharides of either plant cell wall or animal glycan origin^6,35^, and to some extent, against simpler carbohydrates. Pathway analysis using KEGG orthologs indicated that enzymes in the *Prevotella* CAZy cluster participated in various carbohydrate degradation pathways, many of them in degradation of pectin. We also identified clusters of CAZy annotated to Firmicutes including *Faecalibacterium*, but the proteomes did not show a particular CAZy profile analogous to *Bacteroides-* and *Prevotella-*CAZy in individuals with high abundance of *Faecalibacterium*. In spite of overlap of CAZy enzymes in *Bacteroides-* and *Prevotella-*CAZy profiles, many of the glycosyl hydrolase (GH) and other CAZy families in heatmaps were distinct, and principal component analysis of CAZy enzyme intensities identified samples with *Prevotella-*CAZy profile to be unique. This suggests differences between *Bacteroides* and *Prevotella* in their utilization of carbohydrate substrates and that *Prevotella* in these donors were poised towards plant cell wall glycan degradation including xylan degrading enzymes (alpha arabinofuranosidase, xylan 1,4-beta-xylosidase)^6,10,22^.

Increasing fiber intake in diet promotes changes in microbiota composition in the gut, but not in all individuals^36^. A recent study demonstrated donor-dependent increase in *Prevotella copri* in the gut following introduction of barley kernel in the diet, but not in all individuals^12^. In our donors, the average consumption of fiber did not differ between donors with high abundance of *Prevotella* and other donors, not even when compared to those with intermediate levels (> 1%) of *Prevotella* in their microbiota. Their CAZy profile was poised towards enzymes known to participate in foraging of plant-derived glycans and differed from other donors in principal component analysis. This suggests that *Prevotella* in their gut were particular in CAZy genome^5,10,31,35,37^ or in the translation of their certain CAZy genes to enzymes. These differences could translate to different levels of success in competition for niche in the gut, allowing their high abundance in gut without a need for particular fiber intake.

Of note, this study consisted of a cohort of pregnant and overweight women^21^, and as both of these impact on microbiota composition^3,38^, our results may not be directly generalizable to unselected populations. However, donors with lots of *Prevotella* in their microbiota showed no bias in age, BMI, duration of pregnancy nor dietary parameters in comparison with other donors. This suggests that the abundance of *Prevotella* in their microbiota relates either to particular characteristics of their bacteria or to host-related factors not monitored in the cohort.

In summary, by mass-spectrometry based metaproteome analysis we identified two novel CAZy expression profiles in gut microbiota proteome of our donors. These CAZy profiles were mutually exclusive on donor level, included many non-overlapping CAZy enzymes and associated with differences in microbiota composition, suggesting metabolic specialization of individual microbiotas to forage different glycans for energy metabolism. Genomic analyses of *Prevotella* species isolated from oral cavity and gut have revealed considerable strain-to-strain differences in CAZy gene profiles^11^, and expression of CAZy genes are also subject to regulation on transcriptional level^22,30^. Identification of CAZy by metaproteomics provides a possibility to determine metabolic competence of microbiota on a level, which extends metagenomic analyses. Identification of enzyme profiles such as the two reported in this study may pave way to more detailed understanding of the population dynamics of microbiota members.

## Materials and methods

### Samples and study subjects

Study subjects were overweight or obese but otherwise healthy women in their early pregnancy, and their stool samples were originally collected for an intervention trial published earlier^21^. Dietary intake was calculated from three-day-food diaries recorded within a week prior to collection of stool sample. The overall dietary quality was measured by the validated index of diet quality (IDQ) questionnaire that reflects adherence to dietary recommendations^26^. Mean daily intakes of nutrients were calculated using computerized software described earlier^21^.

### Sample preparation for bacterial proteome analyses

For this study, we used parallel stool samples collected at study entry before any intervention. Samples were put at + 4 °C immediately after their collection and an aliquot of the sample was stored at − 80 °C within hours. Thawed fecal material was dissolved in phosphate buffered saline (PBS) at + 4 °C including protease inhibitor (aprotinin) and allowed to dissolve with gentle agitation. Bulk material was removed by spinning the samples at low G force, and supernatant containing bacteria was collected. Bacteria content in the supernatant was determined by in situ labelling of a 16S-RNA consensus sequence to cover the detection of all eubacteria. Bacteria were counted by flow cytometry using a bacterial staining kit (Thermo Fisher) allowing exclusion of dead cells. Following flow cytometry, an aliquot of supernatant containing 10^8^ bacteria was used to prepare each sample. Bacteria were pelleted down and stored as pellets at − 80 °C until protein isolation. Proteins were extracted from pelleted bacteria using NoviPure Microbial Protein kit (MO BIO Laboratories Inc.) according to manufacturer’s instructions. Protease inhibitors (Pierce Protease Inhibitor Tablets, Thermo Scientific) were added to lysis buffer. Mechanical cell lysis was performed by bead-beating using TissueLyser-device (Qiagen) and two 5 min cycles at 50 Hz. Between cycles samples were placed on ice for 5 min. Protein concentrations were determined by DC Lowry (BioRad) method. Fifty microgram proteins were digested by trypsin using filter aided sample preparation (FASP) method^39^. Peptides were desalted by SepPac C18 96-well plate (Waters), evaporated to dryness and dissolved in 0.1% formic acid. Peptide concentrations were checked with NanoDrop device (Thermo Fisher Scientific), and iRT peptides (Biognosys AG) required for retention time calibration were added to all samples according to manufacturer’s instructions before mass spectrometry (MS) analysis.

### Mass spectrometry

Proteins were identified and quantitated using data independent acquisition (DIA) based MS method. A spectral library was created by analyzing seven pooled samples six times with data dependent acquisition method (DDA). All MS analyses were performed on a Q Exactive HF mass spectrometer (Thermo Fisher Scientific, Bremen, Germany) equipped with a nano-electrospray ionization source and connected to a high performance liquid chromatography (HPLC) system (Easy-nLC1200, Thermo Fisher Scientific).

For DDA analysis 2 µg peptides and for DIA analysis 1 µg peptides were loaded on a C18 column (75 μm × 40 cm, ReproSil-Pur 1.9 μm 120 Å C18-AQ, Dr. Maisch HPLC GmbH, Ammerbuch-Entringen, Germany) with flow rate 200 µl/min. The mobile phase consisted of water with 0.1% formic acid (solvent A), or acetonitrile/water (80:20 (v/v)) with 0.1% formic acid (solvent B). A 75 min gradient from 7 to 25% B, followed by 15 min from 25 to 35% B was used to elute peptides.

MS data were acquired automatically by Thermo Xcalibur 3.1 software (Thermo Fisher Scientific). The DDA method consisted of an Orbitrap MS survey scan of mass range 380–1,200 m/z followed by HCD fragmentation of 20 most intense peptide ions. The DIA MS method covered a mass range from 400 to 1,000 m/z through 40 consecutive windows with isolation width of 15 m/z.

### Bacterial proteome analysis

The mass-spectrometry data were analyzed with Diatools software package as described^19,20^ (Diatools version 1.0 (https://github.com/elolab/diatools) to identify the peptides expressed by fecal microbiota of each donor. The overall protein expression profile was constructed using the integrated gene catalog of the human gut microbiome (IGC)^40^ covering over 9 million human gut microbiota proteins. The database was further annotated with CAZy family and protein product names by matching corresponding IGC and CAZy database (https://www.cazy.org) sequences with Diamond program^41^. In total, we identified 5.8% of the IGC sequences as CAZy. Moreover, to focus on bacterial enzymes participating in carbohydrate metabolism, we also searched the mass spectrometry data directly against the CAZy database (https://www.cazy.org).

### 16S-rRNA gene sequencing

Processing of stool samples for DNA extraction and 16S-rRNA gene sequencing were done as described^32^. Briefly, primers targeted V3 and V4 regions of the 16S RNA gene and amplicons were sequenced using Illumina platform^32^. Raw sequences were processed by using the QIIME software package version 1.9.1^42^ (QIIIME: version 1.9.1 (https://qiime.org/). Operational taxonomic units (OTUs) were identified using open-reference OTU picking protocol and chosen at 97% similarity against the Greengenes database (version gg 13 8).

### Statistical analyses

The statistical analyses were conducted using R software version 4.0.0. (https://www.R-project.org/). For proteomics, the data was transformed using centered log-ratio transformation (CLR) and differentially expressed peptides between groups (samples with below or above median *Bacteroides* relative abundance according to 16S rRNA gene sequencing results) were assessed with ROPECA^43^ using the modified *t* test with False discovery rate (FDR) cut-off set to 0.01 or 0.05. Heatmaps from the intensities of differentially expressed CAZy enzymes were generated using the Pretty Heatmaps R package. For heatmaps (version 1.0.12; https://CRAN.R-project.org/package=pheatmap), hierarchical clustering of the samples (columns) and the CAZy enzymes (rows) was performed using the euclidean distance metric using the CLR transformed data. For analyzing the co-occurrence of CAZy families of specific bacterial genera in the samples, we used the symmetric modification of the ρ metric utilizing the centered log ratio -transformed data as described^43^. Similarly, this test was also used for two-parameter comparisons between bacterial abundances derived from the 16S rRNA gene sequencing data.

Principal component analyses (PCA) on microbiota composition and CAZy expression were performed using the prcomp R function on the CLR transformed data. Furthermore, differences in the microbiota composition and CAZy expression between selected donors were assessed by permutational multivariate analysis of variance (PERMANOVA) using the adonis2 function from the vegan R package (https://cran.r-project.org/web/packages/vegan/index.htm). Differences in intake of energy and in dietary variables between donors were analyzed by Mann–Whitney *U* test.

### Ethical considerations

The Ethics Committee of the Hospital District of Southwest Finland approved the clinical study protocol and all participants provided written informed consent for provision of stool sample. The study complies with the Declaration of Helsinki as revised in 2000.

## Supplementary information

Supplementary Information.

## Data Availability

The mass-spectrometry datasets generated during and/or analyzed during the current study are available in the PRIDE (ProteomeXchange) repository. **Project Name:** Correlation of *Prevotella* abundance with a particular carbohydrate-active CAZy enzyme profile in healthy gut microbiota. **Project accession:** PXD017059 **Project DOI:** Not applicable Reviewer account details: **Username:** reviewer53498@ebi.ac.uk. **Password:** ZuHDclhX. 16S-RNA parallel sequencing data is available at https://doi.org/10.5281/zenodo.3608655.
