# Supplementary material for: A carbohydrate-active enzyme (CAZy) profile links successful metabolic specialization of Prevotella to its abundance in gut microbiota
Source: Sci Rep. 2020 Jul 24;10:12411. doi: 10.1038/s41598-020-69241-2 (PMC7381632; doi:10.1038/s41598-020-69241-2)
Supplement: Supplementary file 1 — Supplementary Information. [file 41598_2020_69241_MOESM1_ESM.docx]

**Supplementary information**

**A carbohydrate-active enzyme (CAZy) profile links successful metabolic adaptation**

**of *Prevotella* to its abundancy in gut microbiota**

Juhani Aakko^1^, Sami Pietilä^1,2^, Raine Toivonen^2^, Anne Rokka^1^, Kati Mokkala^2^, Kirsi Laitinen^2^, Laura Elo^1^ and Arno Hänninen^2,3^

From **^1^**Turku Bioscience Centre, University of Turku and Åbo Akademi University, **^2^**Institute of Biomedicine, University of Turku, and **^3^**Department of Clinical Microbiology and Immunology, Turku University Hospital; Turku, Finland.

**Corresponding Author**

Arno Hänninen, MD, PhD

Medisiina D 7022, Kiinamyllynkatu 10

20520 Turku, Finland

[arno.hanninen@utu.fi](mailto:arno.hanninen@utu.fi)

[arno.hanninen@tyks.fi](mailto:arno.hanninen@tyks.fi)

**Suppl. Table 1**.


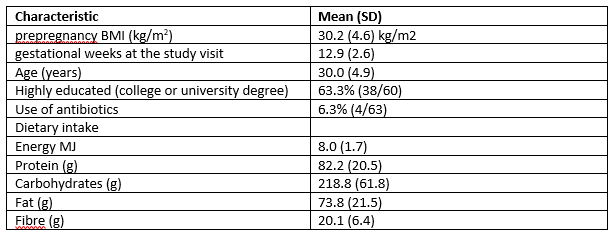


**Suppl. Table 1.** Demographic characteristics and dietary intake of energy and energy-yielding nutrients of the study population. Dietary intake was determined from three-day-food diaries recorded by the women in the week before donating the stool sample. Mean daily intakes of energy and energy-yielding nutrients were calculated using computerized software, Aivo diet 2.0.2.3 (Aivo, Turku Finland). Values are presented as mean (+SD) or as percentages (+range).

**Suppl. Table 2**.


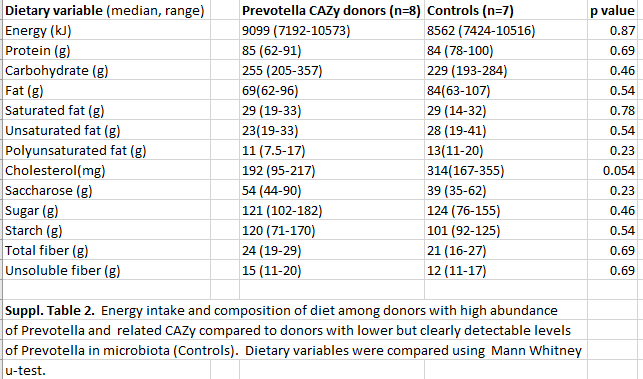


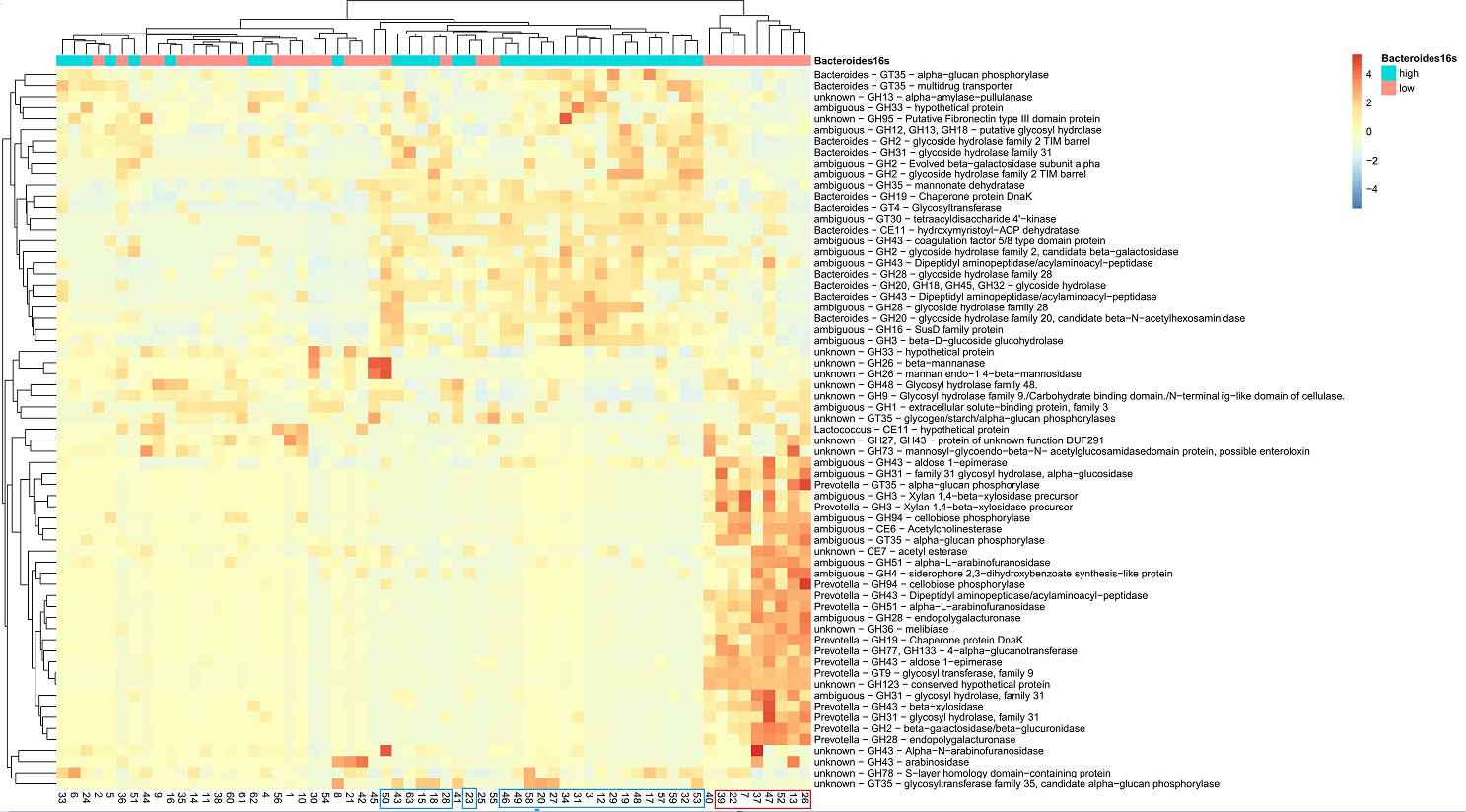


**Supplementary Figure 1**. A list of CAZy enzymes differentially expressed in samples with high abundance of *Bacteroides* in microbiota. For this heatmap, enzymes differentially expressed at FDR < 0.05 were included. This heatmap identifies CAZy expressed differentially in the same groups of donors as in Figure 3b. Donors are framed in this heatmap similarly to Figure 3b.


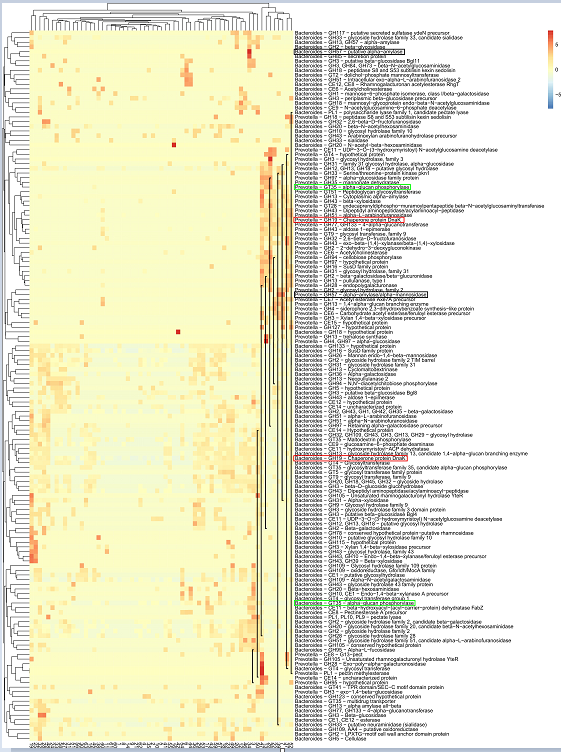


**Suppl. Figure 2.** A list of CAZy with unambiguous *Prevotella* or *Bacteroides* –annotation in the form of a heatmap. Inclusion of CAZy in the list is based on peptide annotations without any profiling of donors. *Prevotella-* and *Bacteroides*- annotated enzymes which are identical are framed and color-coded (black, green and red frames). *Prevotella-* and *Bacteroides*- annotated enzymes which represent the same CAZy family but are *not* identical are marked with straight lines in the heatmap (10 lines with endpoints at corresponding horizontal levels which identify enzyme names).

**Sample # and corresponding sample names in original data files** (see next page)

(The list is the same as in Related Manuscript File 1)

| Sample # in Figures | Metaproteomics Sample Name | 16S SampleName |
| --- | --- | --- |
| 1 | 1 | 45 |
| 2 | 2 | 1 |
| 3 | 3 | 2 |
| 4 | 5 | 47 |
| 5 | 6 | 3 |
| 6 | 7 | 48 |
| 7 | 8 | 4 |
| 8 | 25 | 58 |
| 9 | 27 | 57 |
| 10 | 28 | 11 |
| 11 | 29 | 12 |
| 12 | 31 | 60 |
| 13 | 33 | 98 |
| 14 | 34 | 14 |
| 15 | 35 | 62 |
| 16 | 36 | 63 |
| 17 | 37 | 64 |
| 18 | 39 | 15 |
| 19 | 41 | 16 |
| 20 | 42 | 66 |
| 21 | 43 | 67 |
| 22 | 44 | 68 |
| 23 | 45 | 17 |
| 24 | 46 | 18 |
| 25 | 47 | 19 |
| 26 | 49 | 20 |
| 27 | 50 | 71 |
| 28 | 51 | 21 |
| 29 | 53 | 22 |
| 30 | 57 | 75 |
| 31 | 58 | 76 |
| 32 | 59 | 23 |
| 33 | 60 | 25 |
| 34 | 61 | 77 |
| 35 | 62 | 78 |
| 36 | 63 | 26 |
| 37 | 65 | 28 |
| 38 | 66 | 29 |
| 39 | 68 | 30 |
| 40 | 69 | 31 |
| 41 | 71 | 83 |
| 42 | 73 | 33 |
| 43 | 74 | 34 |
| 44 | 75 | 35 |
| 45 | 76 | 85 |
| 46 | 77 | 86 |
| 47 | 78 | 87 |
| 48 | 79 | 36 |
| 49 | 80 | 88 |
| 50 | 81 | 38 |
| 51 | 82 | 39 |
| 52 | 83 | 40 |
| 53 | 84 | 90 |
| 54 | 85 | 91 |
| 55 | 86 | 41 |
| 56 | 87 | 92 |
| 57 | 88 | 42 |
| 58 | 89 | 43 |
| 59 | 90 | 93 |
| 60 | 91 | 94 |
| 61 | 92 | 44 |
| 62 | 30 | 13 |
| 63 | 32 | 61 |
